# Supplementary material for: On the role of history-dependent adsorbate distribution and metastable states in switchable mesoporous metal-organic frameworks
Source: Nat Commun. 2023 Jun 3;14:3223. doi: 10.1038/s41467-023-38737-6 (PMC10239506; doi:10.1038/s41467-023-38737-6)
Supplement: Supplementary file 1 — Supplementary Information [file 41467_2023_38737_MOESM1_ESM.pdf]

Supplementary Information

On the role of history-dependent adsorbate distribution and metastable states in switchable mesoporous metal-organic frameworks

Walenszus et al.

Supplementary Figures

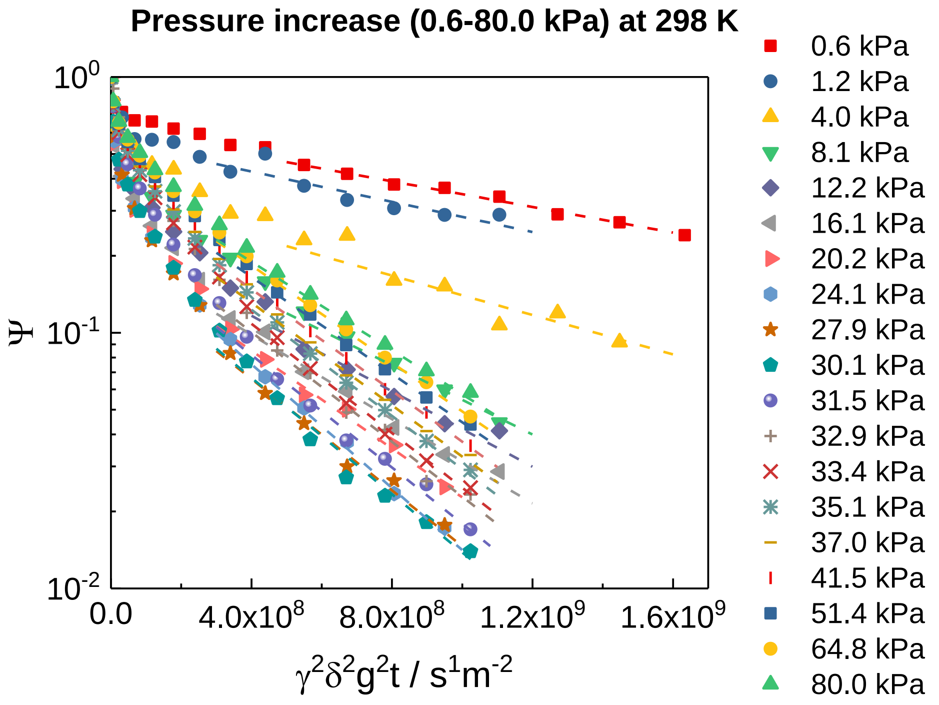

Supplementary Figure S1: Diffusion attenuations acquired for n-butane in DUT-149(Cu) at 298 K during pressure increase from 0.6 kPa to 80 kPa. The conditions are described in the legend.

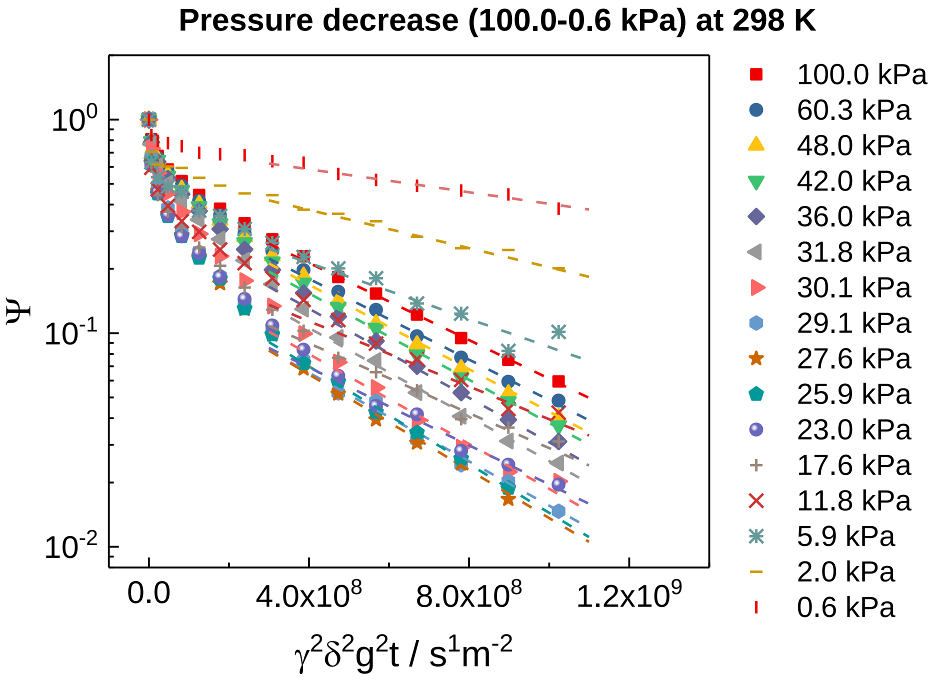

Supplementary Figure S2: Diffusion attenuations acquired for n-butane in DUT-149(Cu) at 298 K during pressure decrease from 100.0 kPa to 0.6 kPa. The conditions are described in the legend.

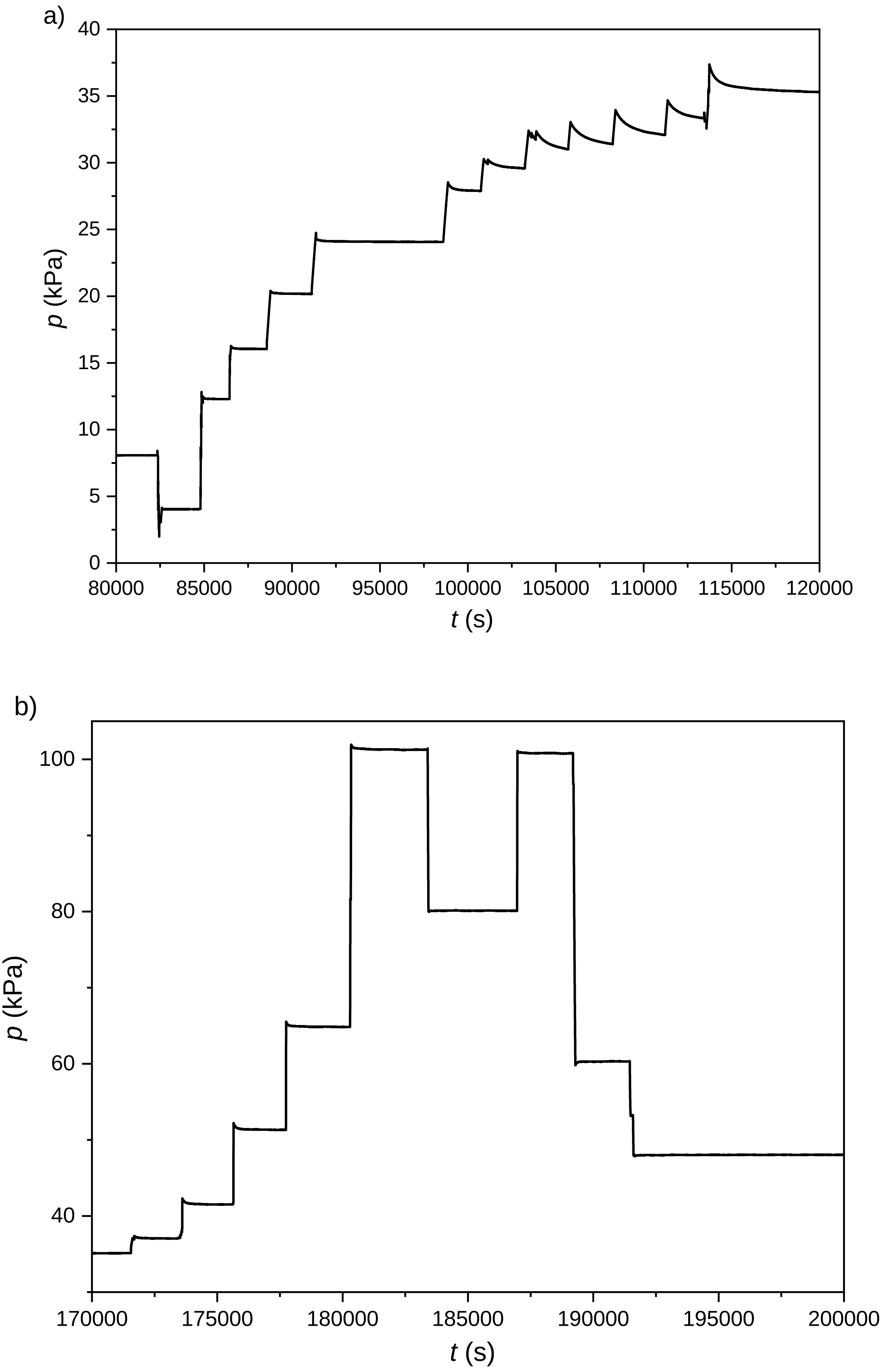

Supplementary Figure S3: Pressure profile of n-butane in the PFG measurement cell, recorded during in situ PFG NMR experiment: a) pressure range 0 – 35 kPa; b) pressure range 35 – 100 kPa.

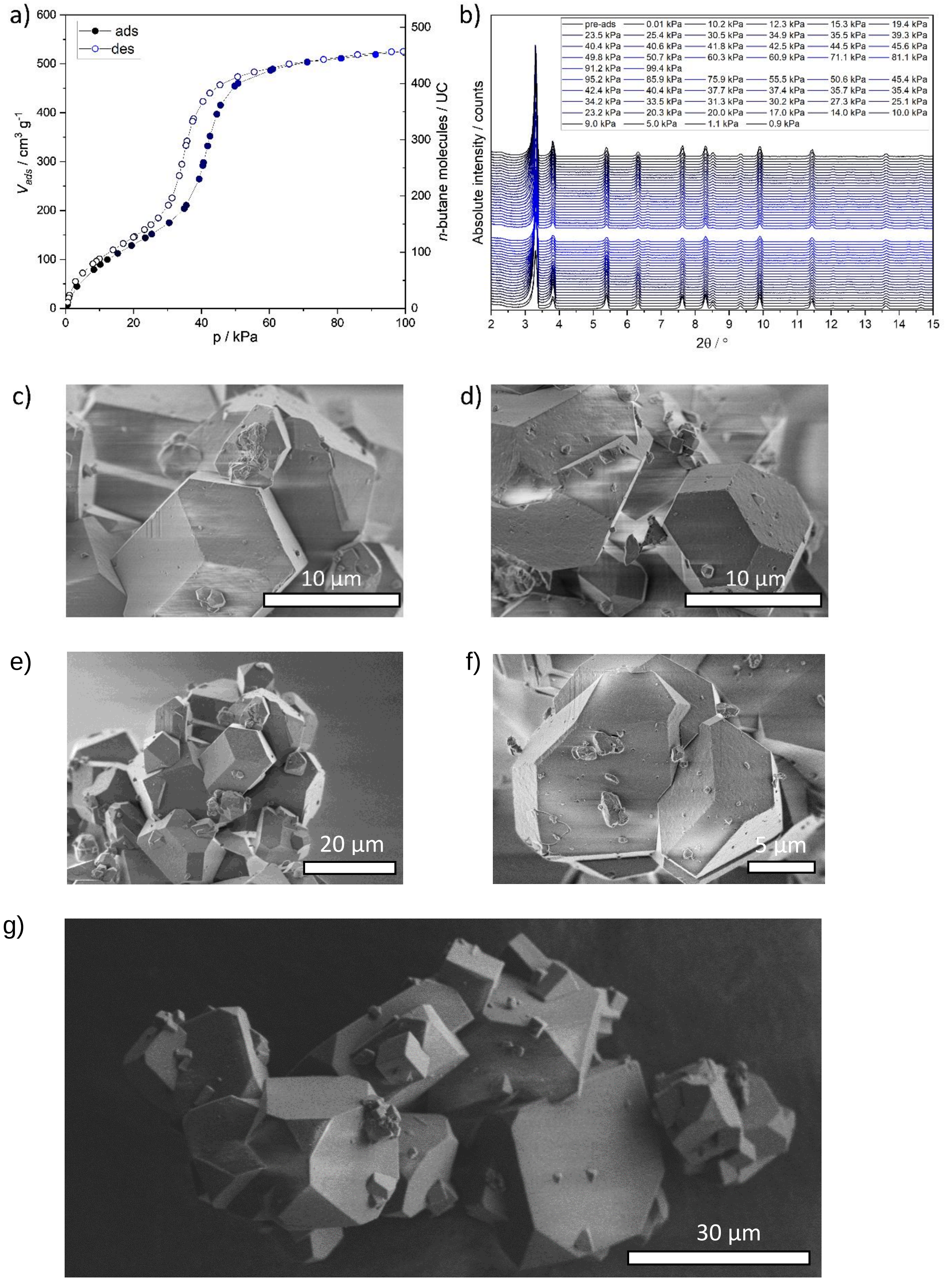

Supplementary Figure S4: In situ PXRD patterns (b), measured on DUT-149(Cu) in parallel to *n*-butane physisorption at 298 K (a) and SEM images, acquired before (c) and after (d-g) experiment.

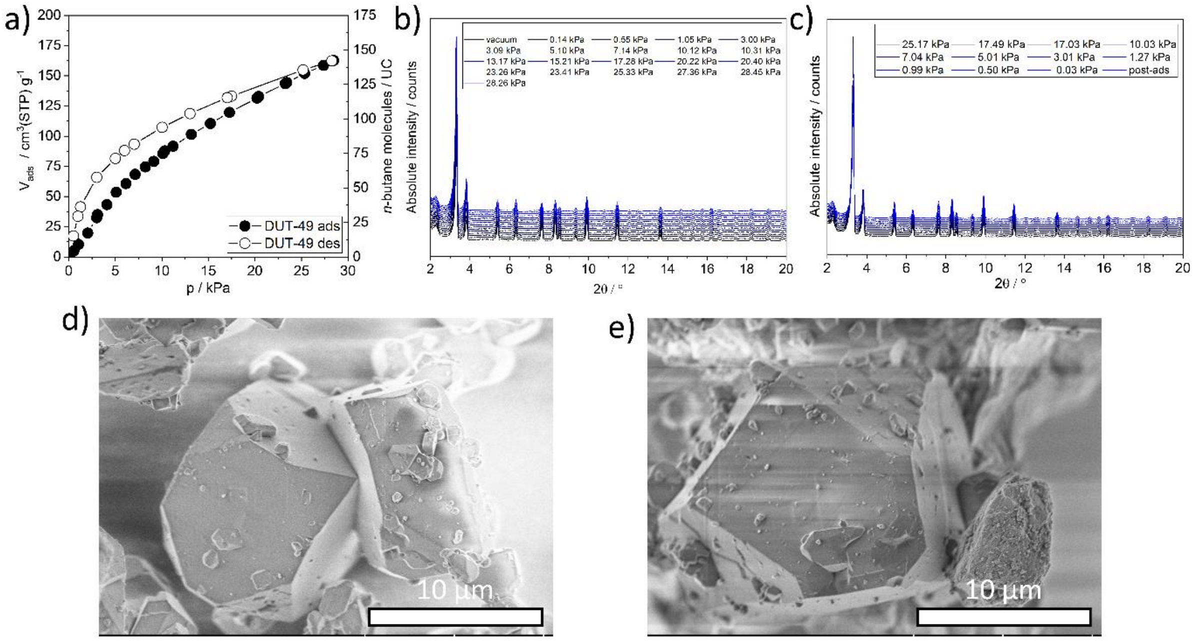

Supplementary Figure S5: In situ PXRD patterns (b), measured on DUT-49(Cu) in parallel to *n*-butane physisorption at 298 K (a) and SEM images, acquired before (c) and after (d) experiment.

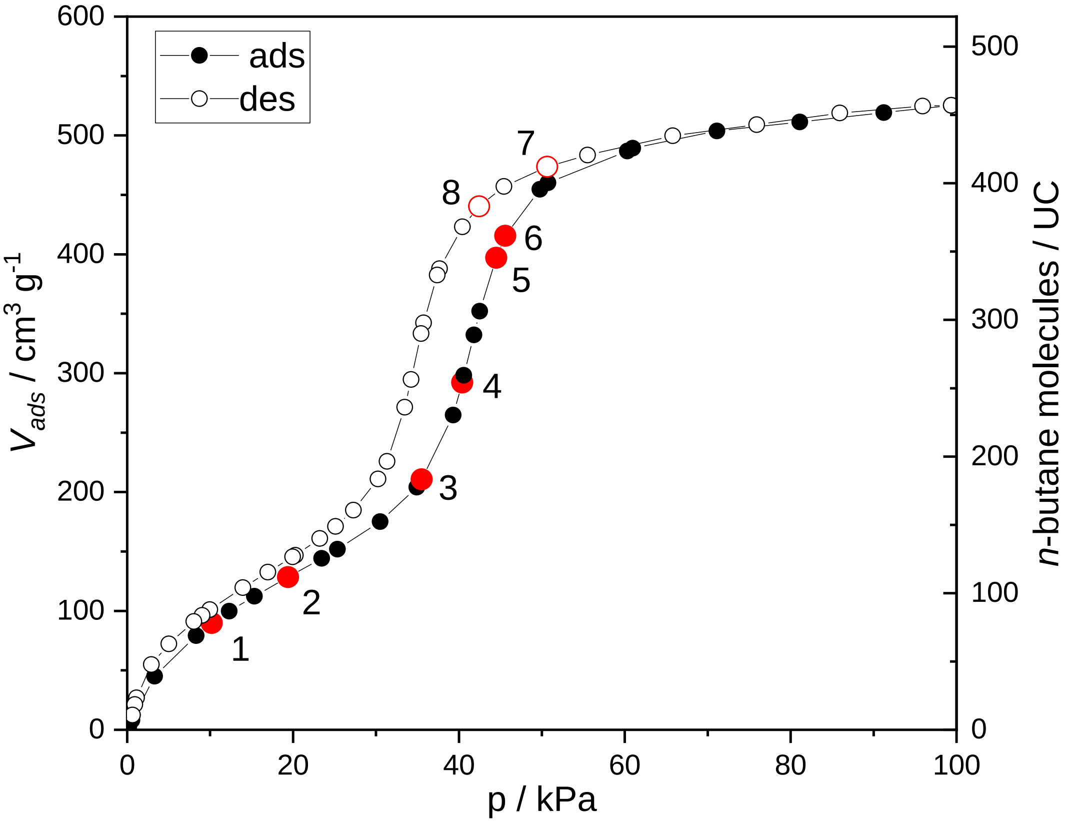

Supplementary Figure S6: Physisorption isotherm of n-butane on DUT-149(Cu) showing selected points, in which the position of n-butane in the pores was determined by Rietveld refinement.

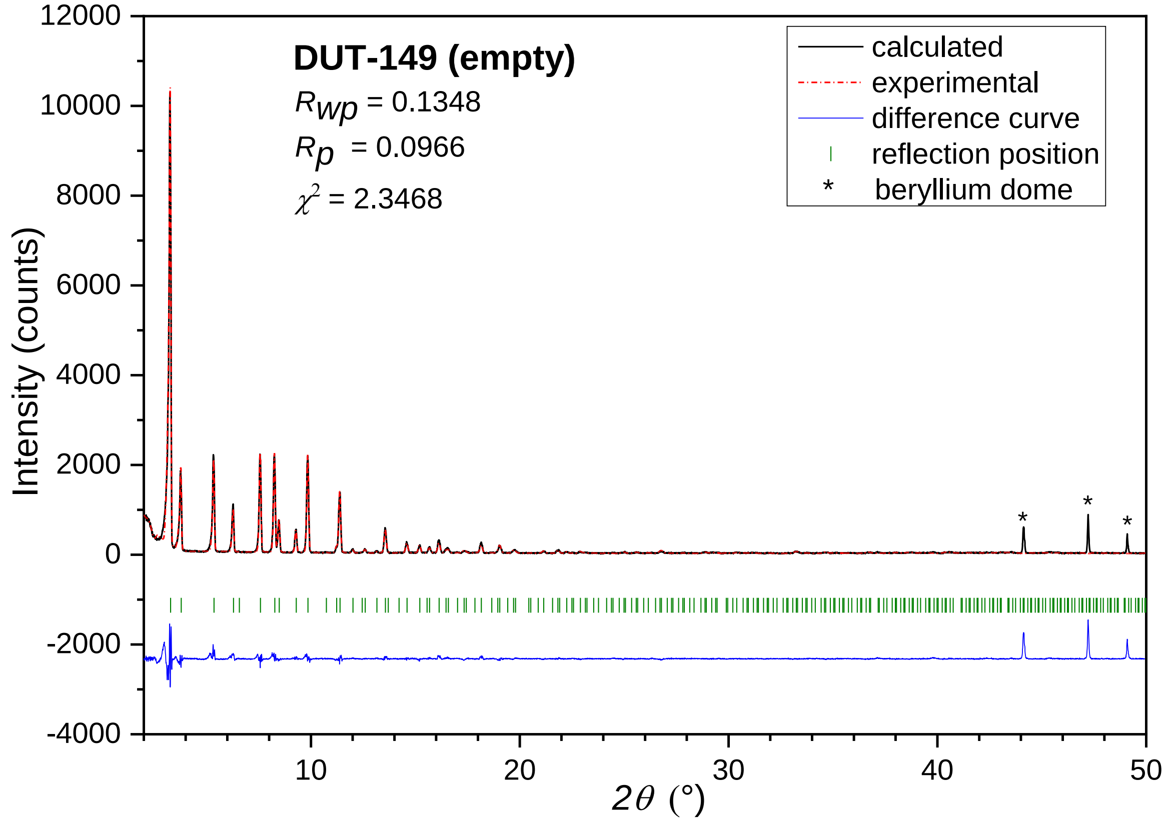

Supplementary Figure S7: Rietveld refinement plot for DUT-149(Cu) guest-free structure.

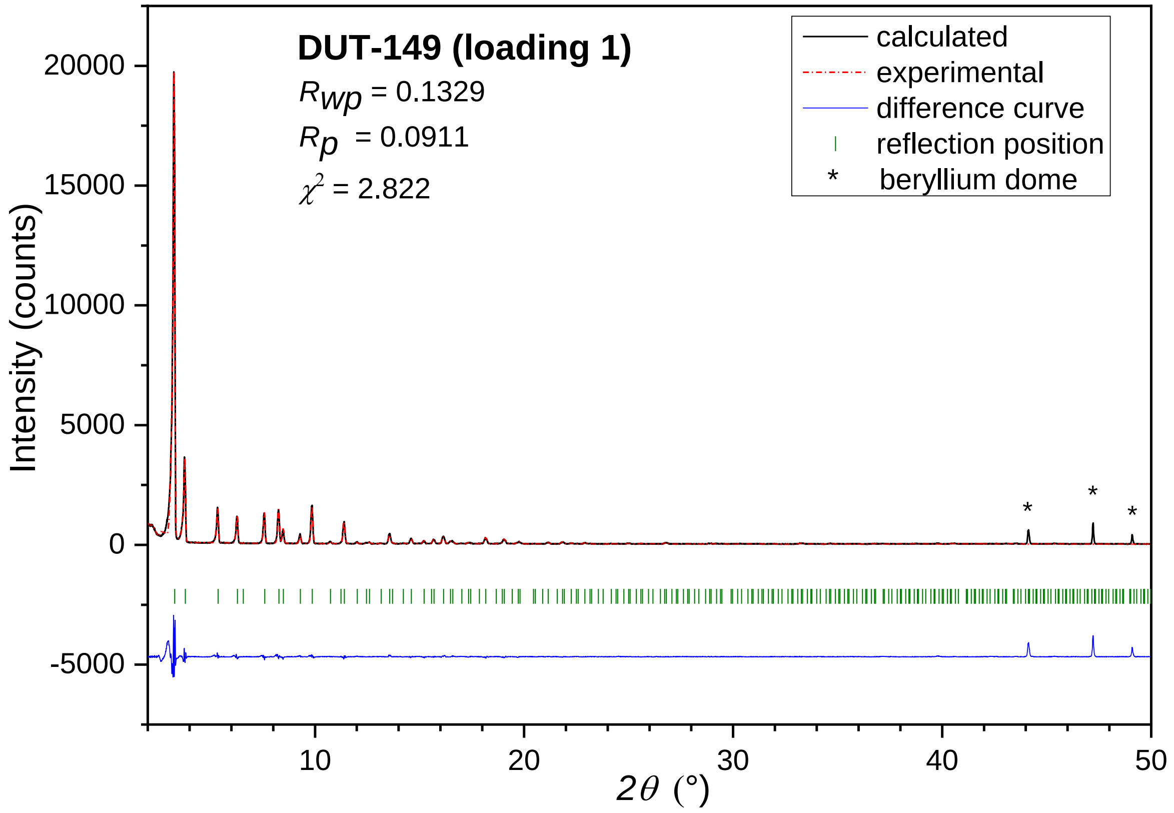

Supplementary Figure S8: Rietveld refinement plot for DUT-149(Cu) loading 1.

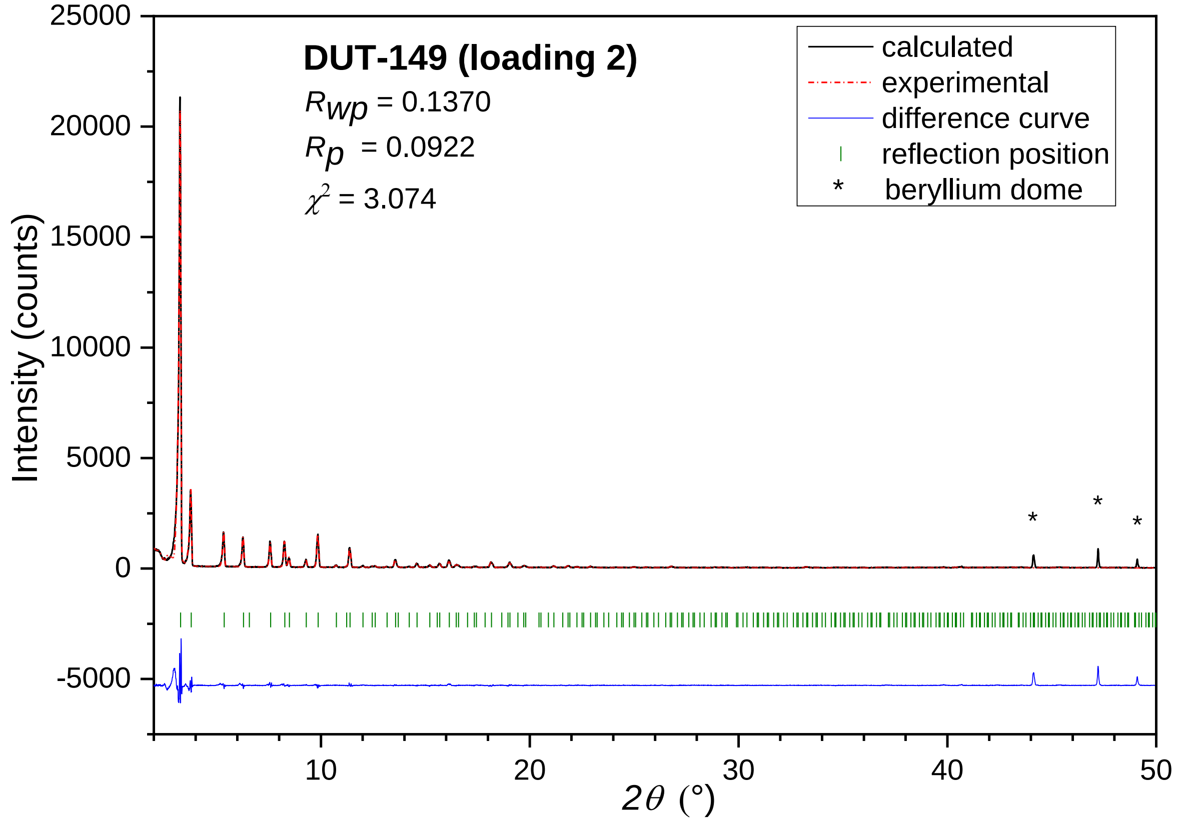

Supplementary Figure S9: Rietveld refinement plot for DUT-149(Cu) loading 2.

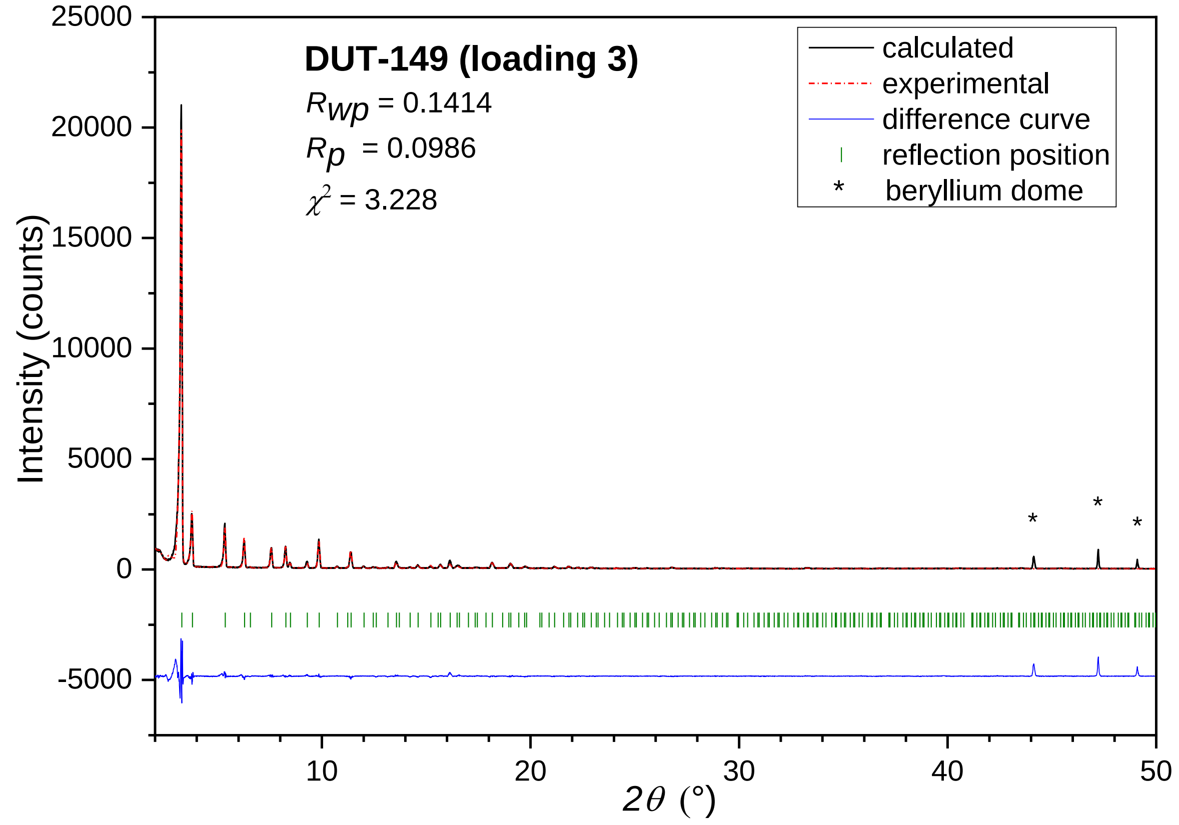

Supplementary Figure S10: Rietveld refinement plot for DUT-149(Cu) loading 3.

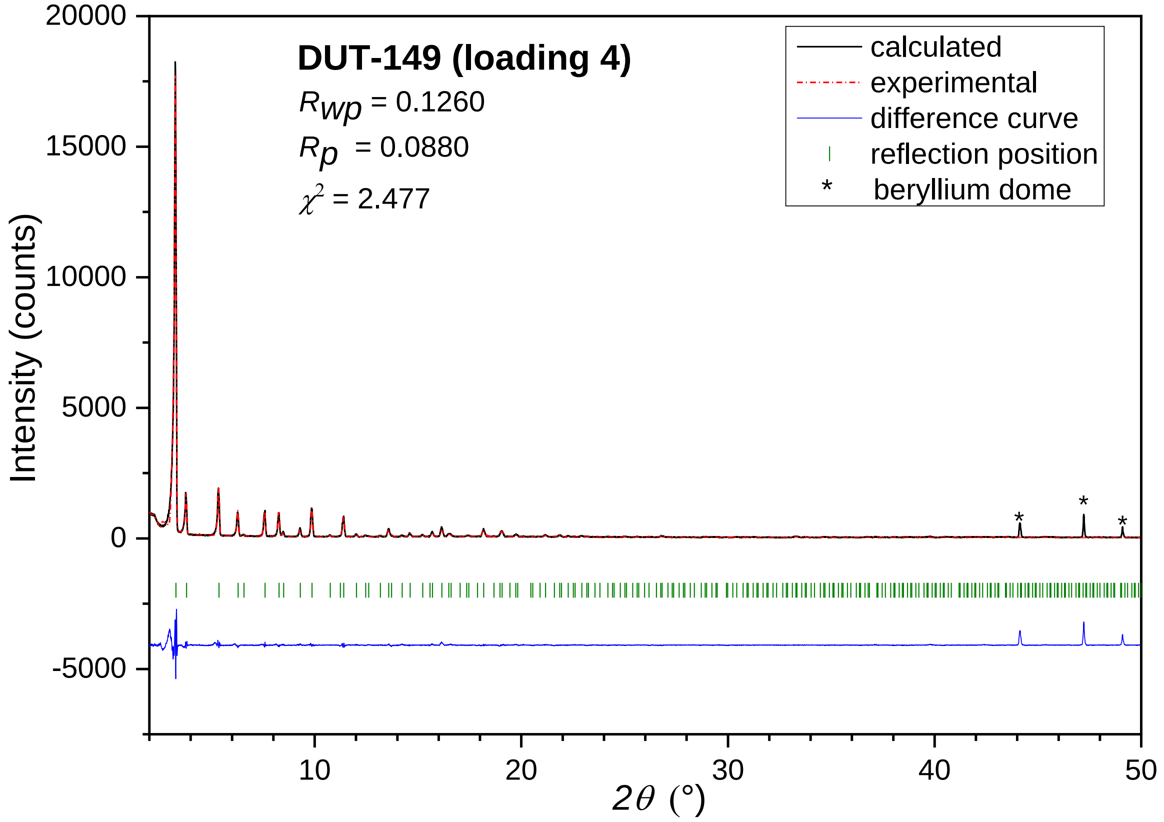

Supplementary Figure S11: Rietveld refinement plot for DUT-149(Cu) loading 4.

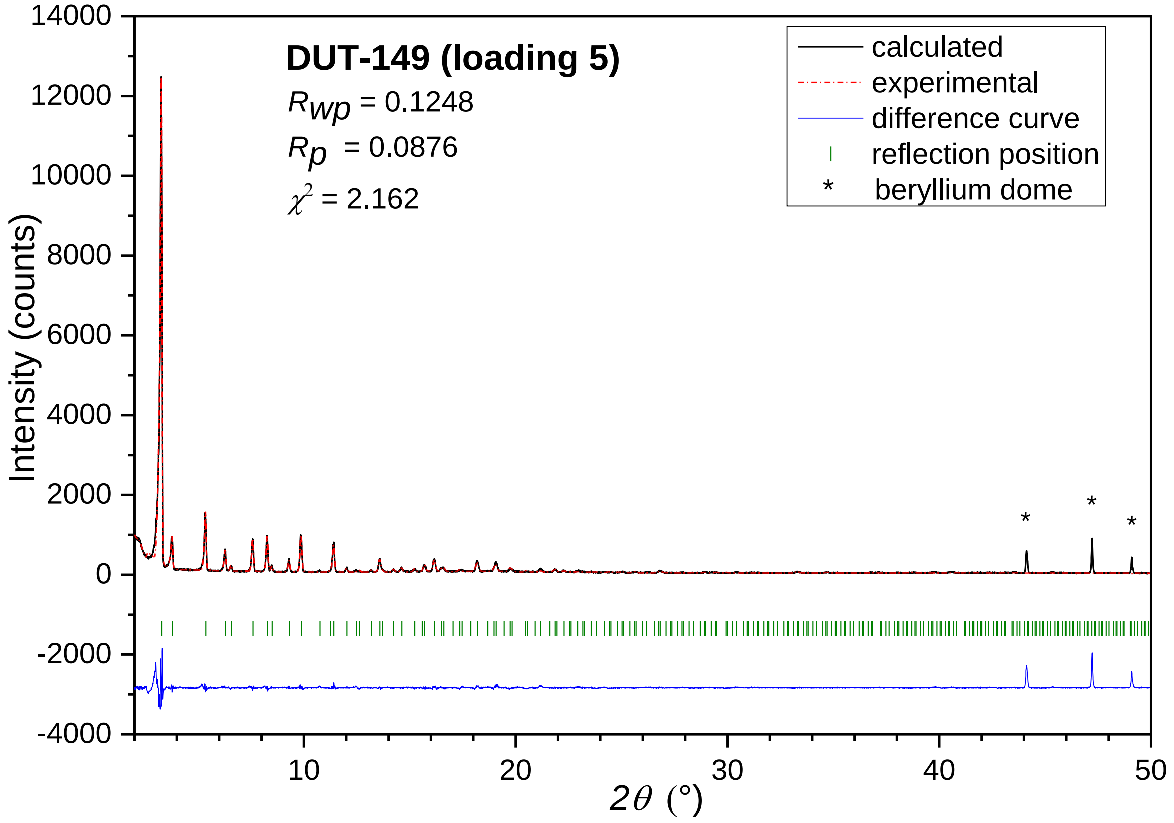

Supplementary Figure S12: Rietveld refinement plot for DUT-149(Cu) loading 5.

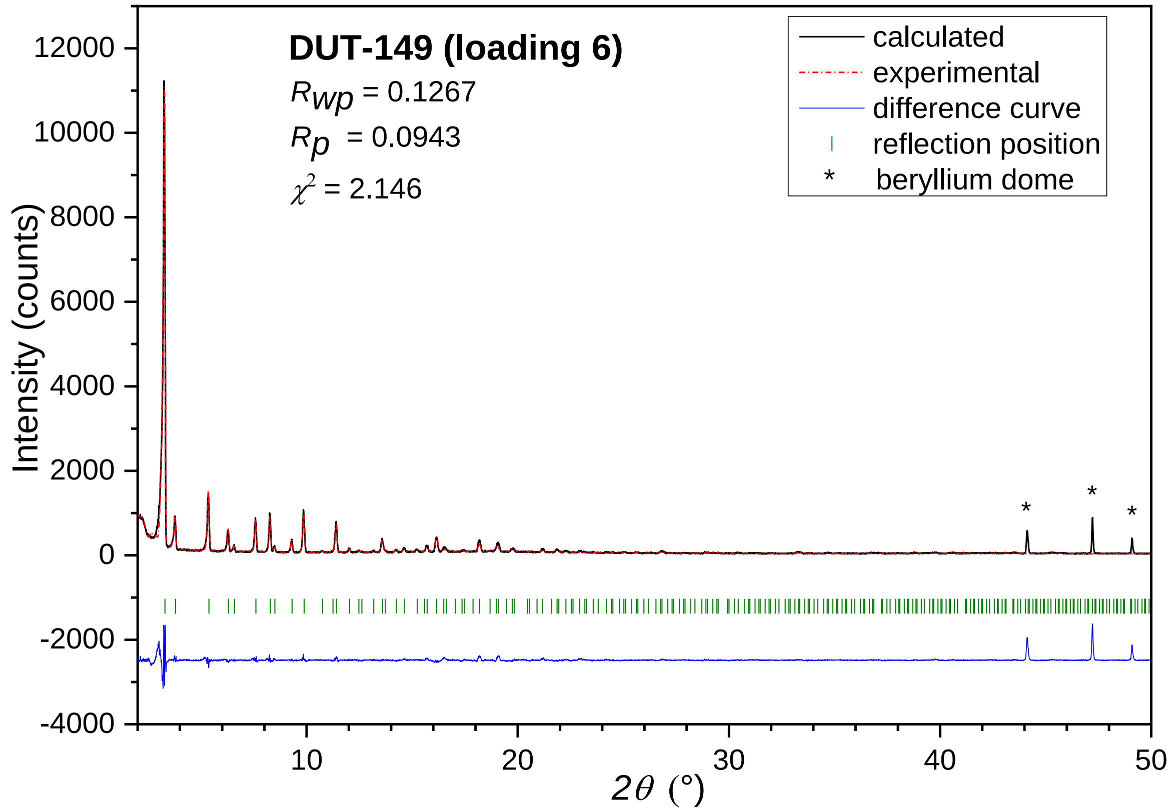

Supplementary Figure S13: Rietveld refinement plot for DUT-149(Cu) loading 6.

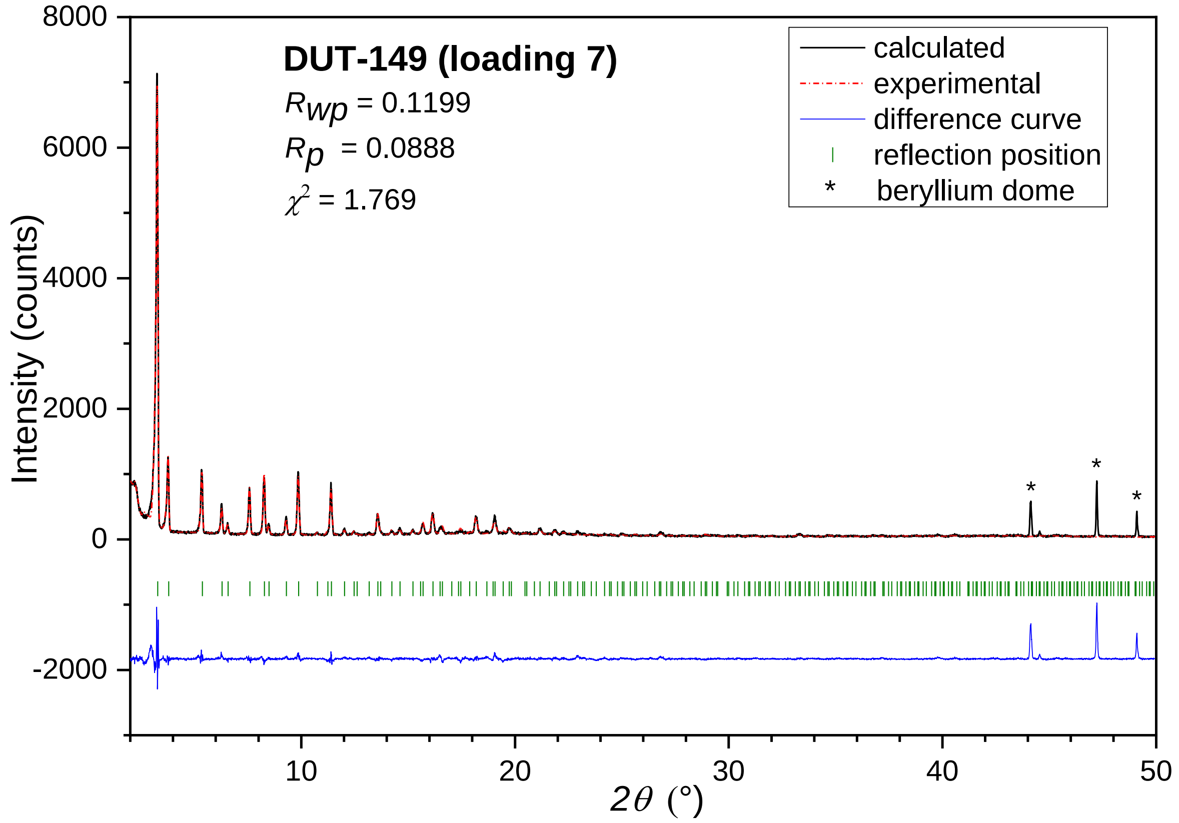

Supplementary Figure S14: Rietveld refinement plot for DUT-149(Cu) loading 7.

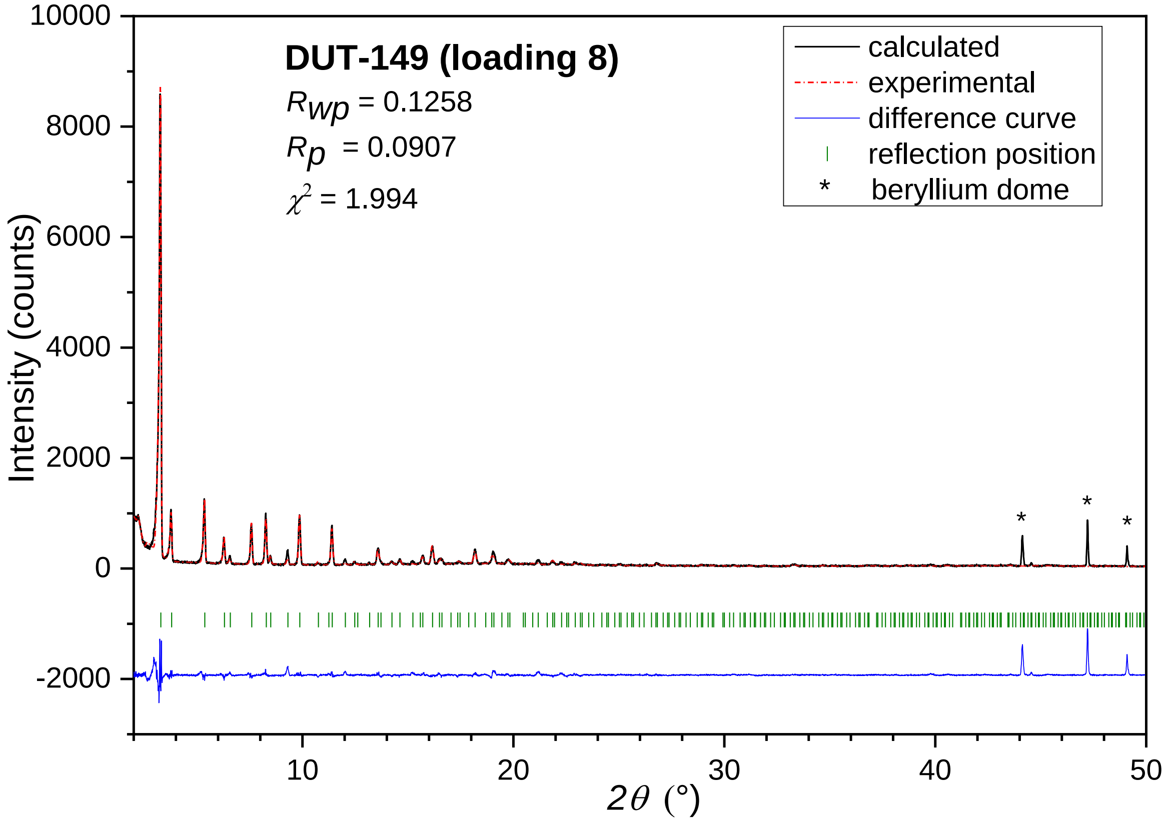

Supplementary Figure S15: Rietveld refinement plot for DUT-149(Cu) loading 8.

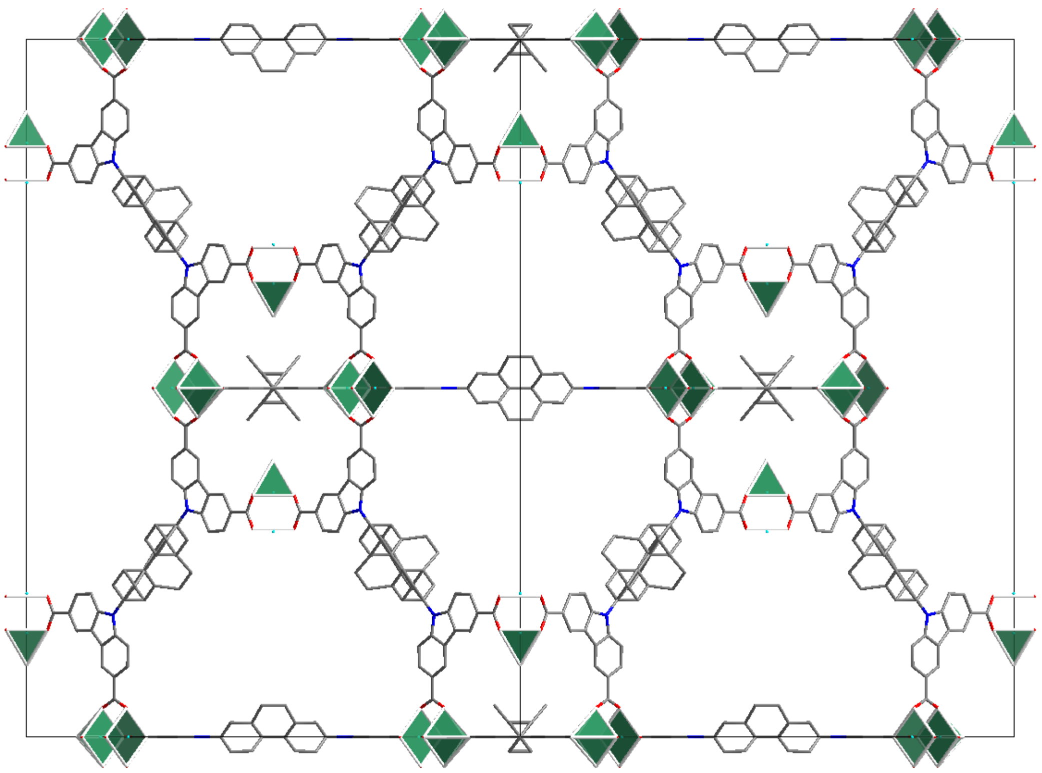

Supplementary Figure S16: Crystal structure of guest-free DUT-149(Cu) framework (projection along [110] direction).

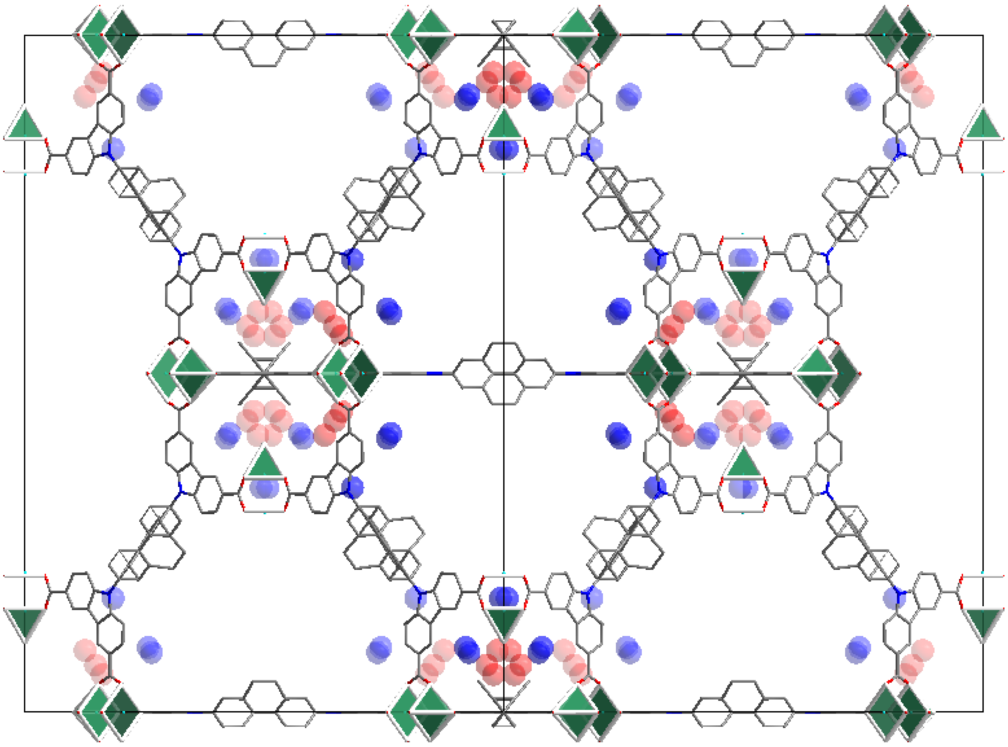

Supplementary Figure S17: Crystal structure of n-butane loaded (loading 1) DUT-149(Cu) framework (projection along [110] direction). Color codes for n-butane adsorption sites: a (red), b (blue), c (green), d (orange), e (purple). The occupancy of the sites is proportional to the transparency of the spheres).

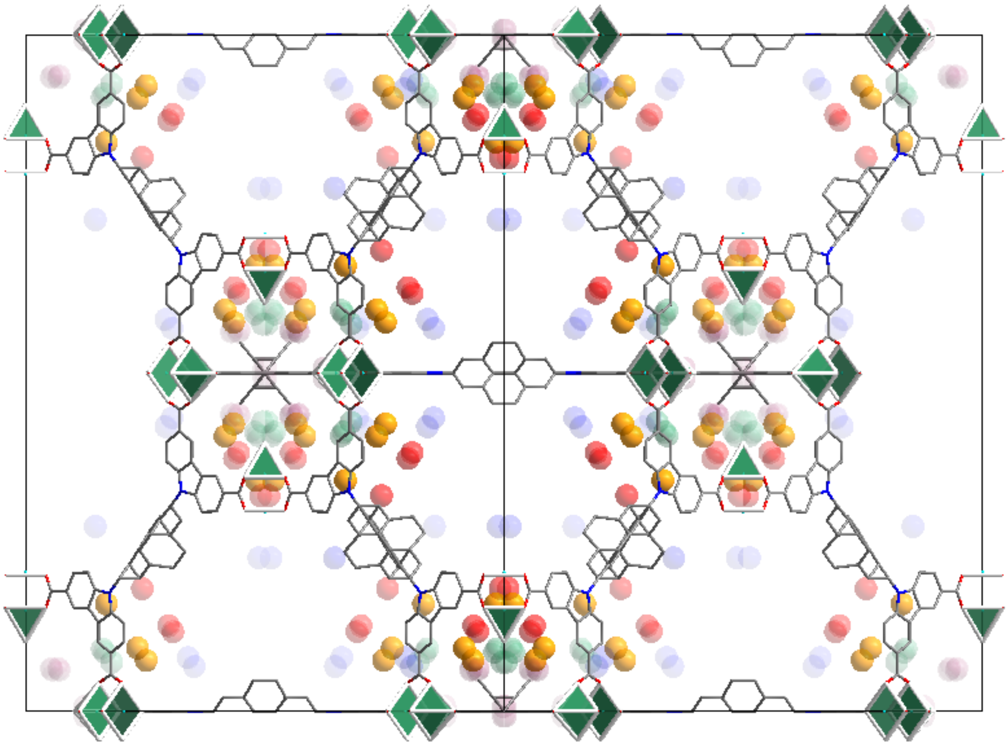

Supplementary Figure S18: Crystal structure of n-butane loaded (loading 2) DUT-149(Cu) framework (projection along [110] direction). Color codes for n-butane adsorption sites: a (red), b (blue), c (green), d (orange), e (purple). The occupancy of the sites is proportional to the transparency of the spheres).

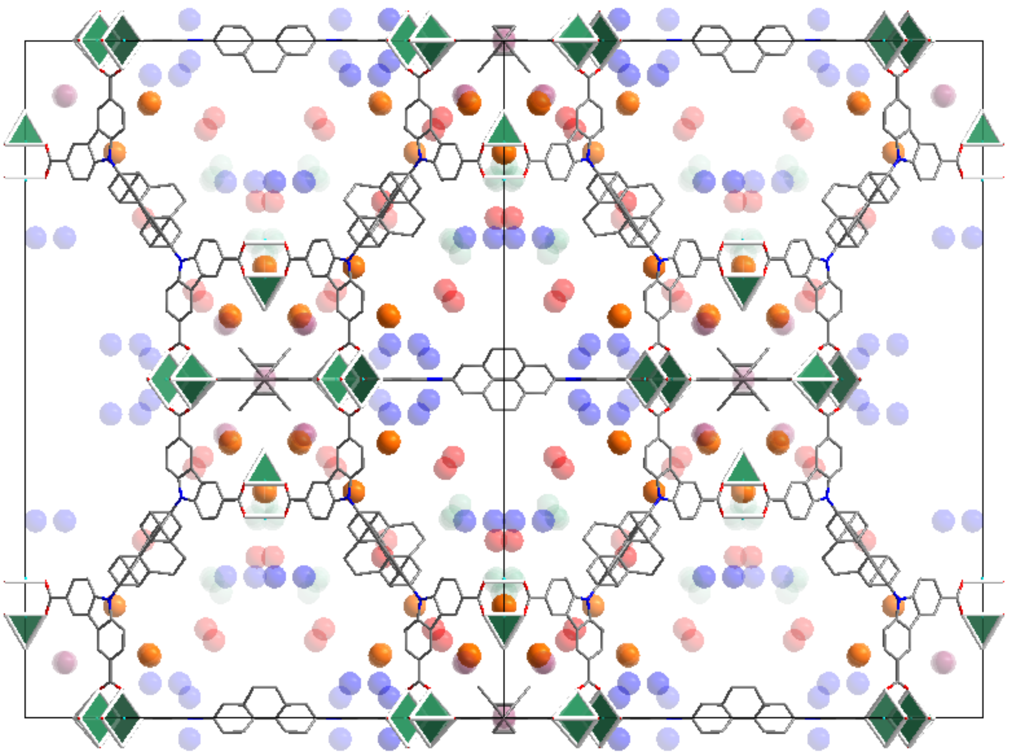

Supplementary Figure S19: Crystal structure of n-butane loaded (loading 3) DUT-149(Cu) framework (projection along [110] direction). Color codes for n-butane adsorption sites: a (red), b (blue), c (green), d (orange), e (purple). The occupancy of the sites is proportional to the transparency of the spheres).

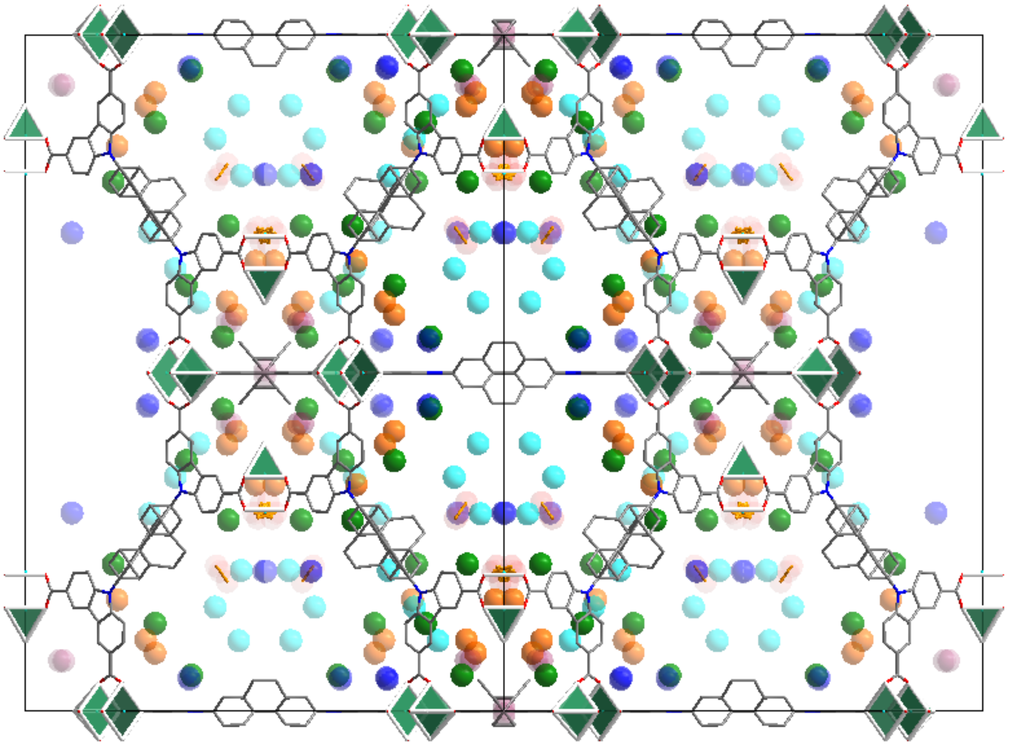

Supplementary Figure S20: Crystal structure of n-butane loaded (loading 4) DUT-149(Cu) framework (projection along [110] direction). Color codes for n-butane adsorption sites: a (red), b (blue), c (green), d (orange), e (purple), f (turquoise). The occupancy of the sites is proportional to the transparency of the spheres).

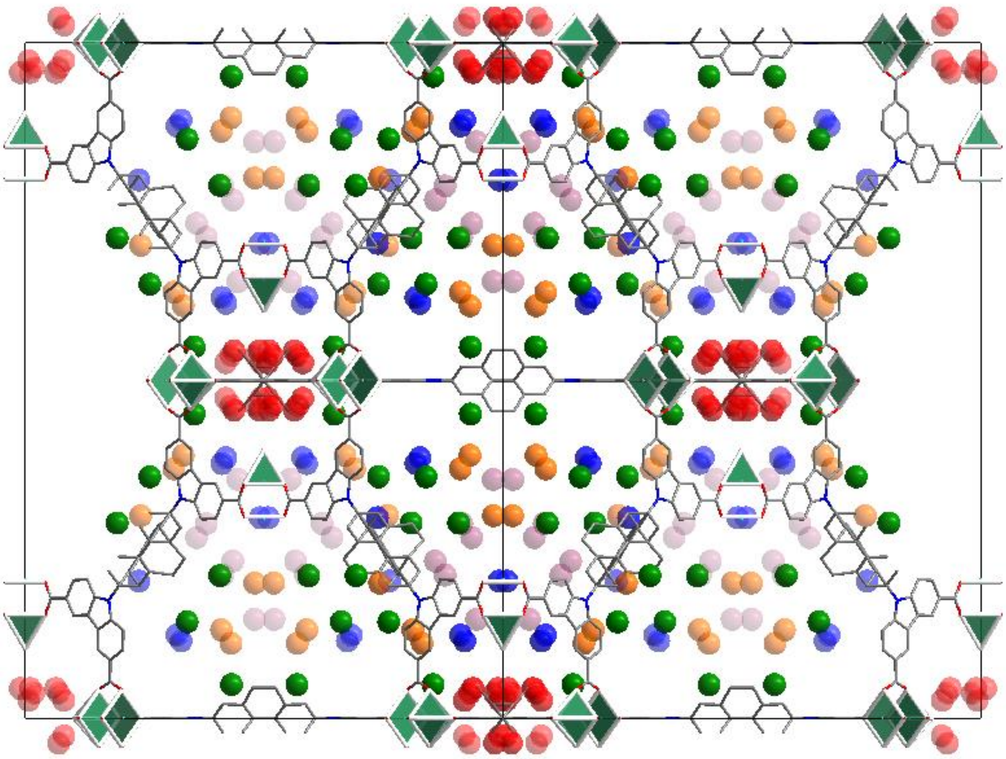

Supplementary Figure S21. Crystal structure of n-butane loaded (loading 5) DUT-149(Cu) framework (projection along [110] direction). Color codes for n-butane adsorption sites: a (red), b (blue), c (green), d (orange), e (purple). The occupancy of the sites is proportional to the transparency of the spheres).

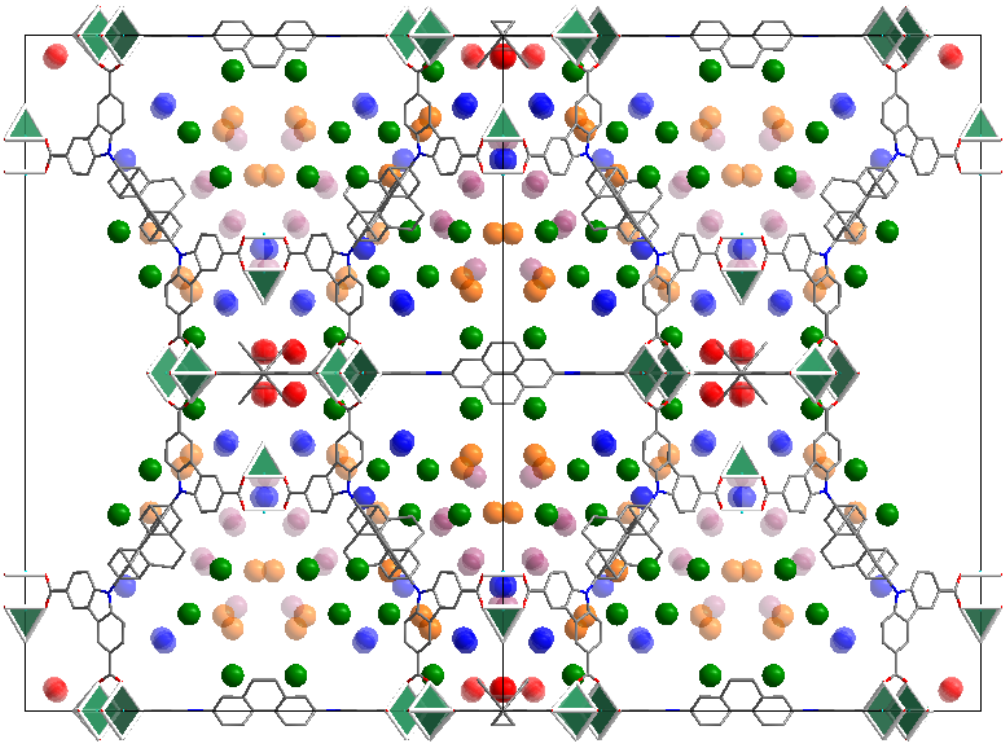

Supplementary Figure S22: Crystal structure of n-butane loaded (loading 6) DUT-149(Cu) framework (projection along [110] direction). Color codes for n-butane adsorption sites: a (red), b (blue), c (green), d (orange), e (purple). The occupancy of the sites is proportional to the transparency of the spheres).

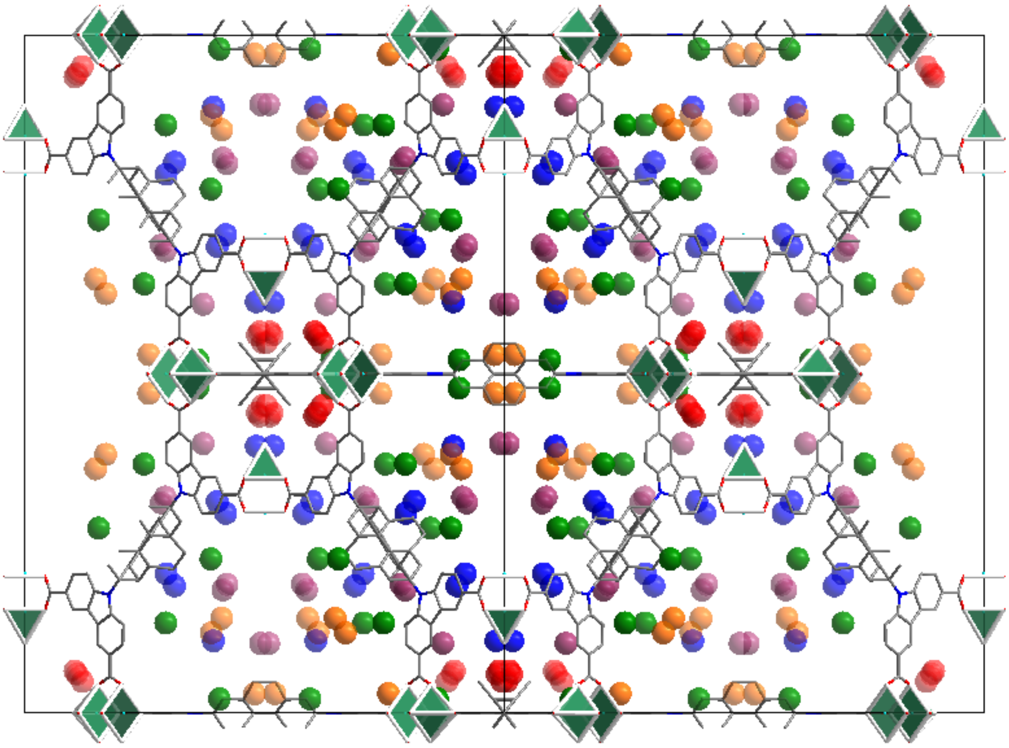

Supplementary Figure S23: Crystal structure of n-butane loaded (loading 7) DUT-149(Cu) framework (projection along [110] direction). Color codes for n-butane adsorption sites: a (red), b (blue), c (green), d (orange), e (purple). The occupancy of the sites is proportional to the transparency of the spheres).

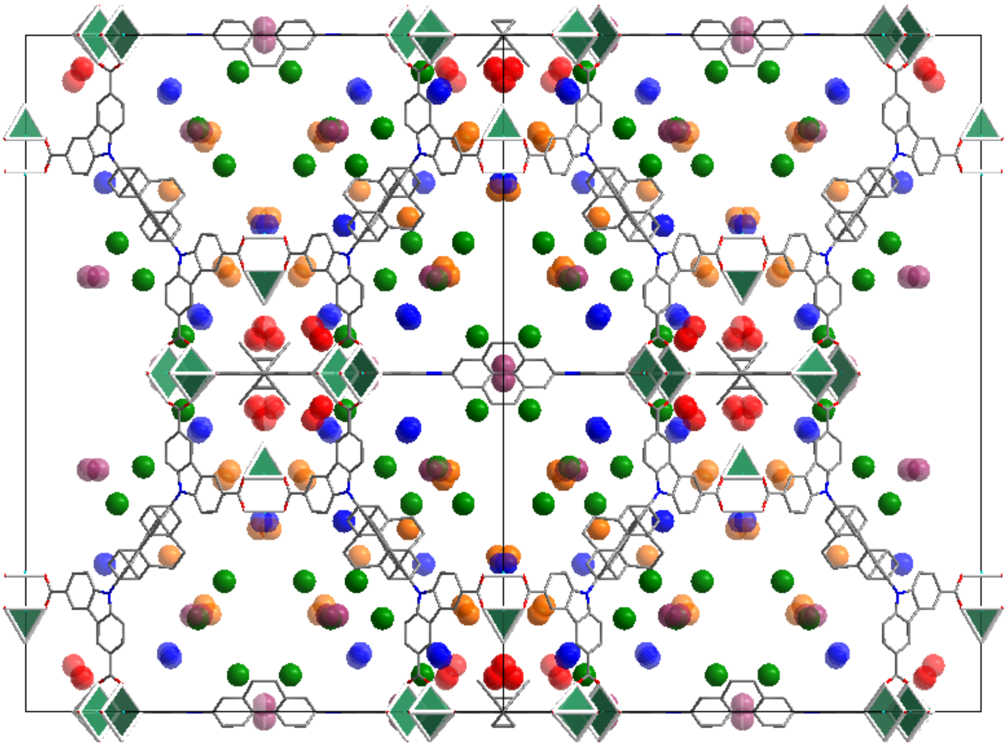

Supplementary Figure S24: Crystal structure of n-butane loaded (loading 8) DUT-149(Cu) framework (projection along [110] direction). Color codes for n-butane adsorption sites: a (red), b (blue), c (green), d (orange), e (purple). The occupancy of the sites is proportional to the transparency of the spheres).

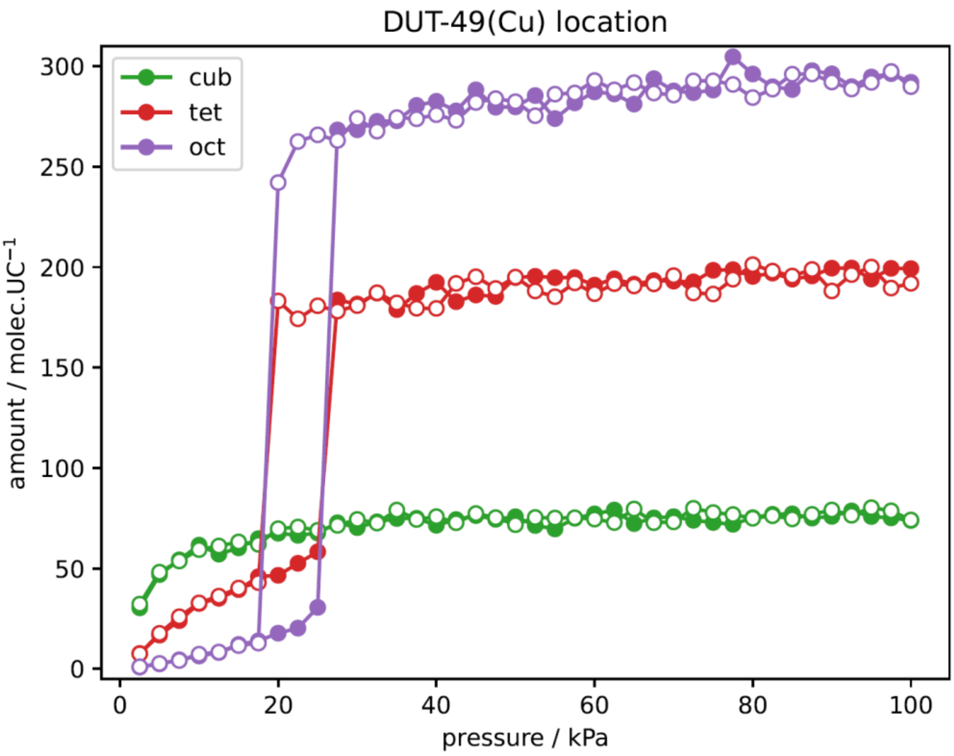

Supplementary Figure S25: n-butane location during adsorption (closed symbols) and desorption (open symbols) at 298 K for the op-state of DUT-49(Cu).

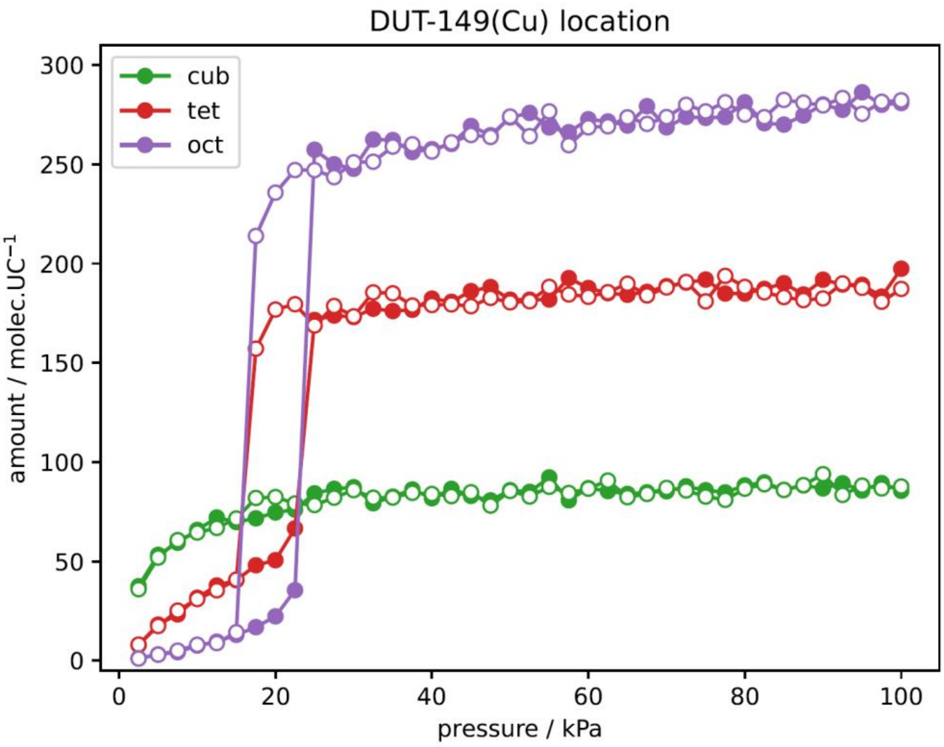

Supplementary Figure S26: n-butane location during adsorption (closed symbols) and desorption (open symbols) at 298 K for the op-state of DUT-149(Cu).

Supplementary Tables

Supplementary Table S1: Resulting self-diffusion coefficients obtained from the fitting of attenuation curves of Figs. S1, S2 and corresponding root mean squared displacements (RMSD)

Pressure increase			Pressure decrease		
p / kPa	D / m^2s^{-1}	RMSD / µm	p / kPa	D / m^2s^{-1}	RMSD / µm
0.6	$5.77 \cdot 10^{-10}$	5.8	100	$2.07 \cdot 10^{-9}$	10.9
1.2	$6.81 \cdot 10^{-10}$	6.3	60.3	$2.18 \cdot 10^{-9}$	11.3
4	$8.87 \cdot 10^{-10}$	7.2	48	$2.28 \cdot 10^{-9}$	11.5
8.1	$1.56 \cdot 10^{-9}$	9.5	42	$2.33 \cdot 10^{-9}$	11.7
12.2	$1.71 \cdot 10^{-9}$	9.9	36	$2.41 \cdot 10^{-9}$	11.8
16.1	$1.90 \cdot 10^{-9}$	10.5	31.8	$2.39 \cdot 10^{-9}$	11.8
20.2	$2.20 \cdot 10^{-9}$	11.3	30.1	$2.42 \cdot 10^{-9}$	11.9
24.1	$2.79 \cdot 10^{-9}$	12.8	29.1	$2.39 \cdot 10^{-9}$	11.8
27.9	$2.51 \cdot 10^{-9}$	12.1	27.6	$2.58 \cdot 10^{-9}$	12.3
30.1	$2.62 \cdot 10^{-9}$	12.3	25.9	$2.63 \cdot 10^{-9}$	12.4
31.5	$2.53 \cdot 10^{-9}$	12.2	23	$2.10 \cdot 10^{-9}$	11.1
32.9	$2.47 \cdot 10^{-9}$	12.0	17.6	$1.88 \cdot 10^{-9}$	10.5
33.4	$2.46 \cdot 10^{-9}$	12.0	11.8	$1.72 \cdot 10^{-9}$	10.0
35.1	$2.39 \cdot 10^{-9}$	11.8	5.9	$1.51 \cdot 10^{-9}$	9.4
37	$2.31 \cdot 10^{-9}$	11.6	2	$1.03 \cdot 10^{-9}$	7.8
41.5	$2.29 \cdot 10^{-9}$	11.6	0.6	$6.20 \cdot 10^{-10}$	6.0
51.4	$2.18 \cdot 10^{-9}$	11.3			
64.8	$2.16 \cdot 10^{-9}$	11.3			
80	$2.03 \cdot 10^{-9}$	10.9			
100	$2.07 \cdot 10^{-9}$	11.0			

Supplementary Table S2: In situ PXRDs during adsorption of n-butane on DUT-149(Cu)

Point	p_e (kPa)	Loading *n*-butane / unit cell (isotherm)	located *n*-butane / unit cell (PXRD)	Position (occupancy) of *n*-butane in the pores
1	10.2	78.14	64.7	a (0.13), b (0.21)
2	19.4	111.78	94.4	a (0.18), b (0.05), c (0.10), d (0.12), e (0.05)
3	35.5	183.26	155.7	a (0.14), b (0.12), c (0.04), d (0.40), e (0.12)
4	40.4	254.16	255.9	a (0.04), b (0.20), c (0.45), d (0.30), e (0.12), f (0.23)
5	44.5	345.44	377.0	a (0.26), b (0.46), c (0.79), d (0.33), e (0.13)
6	45.6	361.48	390.6	a (0.26), b (0.31), c (1.00), d (0.31), e (0.16)
7	50.6	412.06	387.2	a (0.31), b (0.42), c (0.57), d (0.36), e (0.36)
8	42.4	383.15	422.6	a (0.35), b (0.41), c (0.76), d (0.39), e (0.29)

Supplementary Table S3: Experimental data on Rietveld refinement

Point	Unit cell parameter a (Å)	R_{wp}	R_p	χ^2
evac	46.5573(9)	13.4805	9.6644	2.3468
1	46.5380(9)	13.2899	9.1095	2.8220
2	46.5427(10)	13.6987	9.2221	3.0735
3	46.5343(11)	14.1360	9.8625	3.2280
4	46.5066(10)	12.6020	8.8018	2.4770
5	46.4926(6)	12.4778	8.7603	2.1617
6	46.4873(6)	12.6645	9.4285	2.1461
7	46.4991(5)	11.9858	8.8790	1.7694
8	46.4904(11)	12.5842	9.0690	1.9944
