# Supplementary material for: On the role of history-dependent adsorbate distribution and metastable states in switchable mesoporous metal-organic frameworks
Source: Nat Commun. 2023 Jun 3;14:3223. doi: 10.1038/s41467-023-38737-6 (PMC10239506; doi:10.1038/s41467-023-38737-6)
Supplement: Supplementary file 3 — Description of Additional Supplementary Files [file 41467_2023_38737_MOESM3_ESM.docx]

**Description of Additional Supplementary Files**

Supplementary Movie 1

Description: the crystal structure of DUT-49 showing five adsorption sites of *n*-butane in DUT-49 at *p_e_* = 35.5 kPa or 183 mol. / u.c.

Supplementary Movie 2

Description: the crystal structure of DUT-49 showing five adsorption sites of *n*-butane in DUT-49 at *p_e_* = 35.5 kPa or 183 mol. / u.c.
